# Can Fiber Application Affect the Fracture Strength of Endodontically Treated Teeth Restored with a Low Viscosity Bulk-Fill Composite?

**DOI:** 10.1155/2019/3126931

**Published:** 2019-01-22

**Authors:** Evrim Eliguzeloglu Dalkılıç, Magrur Kazak, Duygu Hisarbeyli, Mehmet Ali Fildisi, Nazmiye Donmez, Hacer Deniz Arısu

**Affiliations:** ^1^Bezmialem Vakıf University Faculty of Dentistry, Department of Restorative Dentistry, Istanbul, Turkey; ^2^Gazi University Faculty of Dentistry, Department of Restorative Dentistry, Ankara, Turkey

## Abstract

**Objective:**

The aim of this study is to evaluate the effects of different fiber insertion techniques and thermomechanical aging on the fracture resistance of endodontically treated mandibular premolar teeth restored using bulk-fill composites.

**Materials and Methods:**

Eighty human mandibular premolar teeth were randomly divided into eight groups: Group IN, Group BF, Group PRF1, Group PRF2, Group IN-TMA, Group BF-TMA, Group PRF1-TMA ,and Group PRF2-TMA. Group IN (intact) and Group IN-TMA (intact but subjected to thermomechanical aging) served as control groups. In the other six groups, endodontic treatment was performed and standardized mesio-occluso-distal (MOD) cavities were prepared. In BF, PRF1, and PRF2, the cavities were restored with bulk-fill composite only, bulk-fill/Ribbond, and bulk-fill/additional Ribbond, respectively. In BF-TMA, PRF1-TMA, and PRF2-TMA, the teeth were subjected to thermomechanical aging after the restorations. All of the teeth were fractured on the universal testing machine. Fracture surfaces were analyzed with a stereomicroscope.

**Results:**

Control groups showed significantly higher fracture strengths than tested groups (*P*<0.05). No statistically significant difference was observed among the tested groups (*P*>0.05). Most of the favorable fractures were seen in PRF1, PRF2, and PRF2-TMA. Most of the unfavorable fractures were seen in BF-TMA.

**Conclusions:**

Although fiber insertion with different techniques did not increase the fracture strength of teeth restored with bulk-fill composites, it increased the favorable fracture modes. Thermomechanical aging did not change the fracture strength of the groups.

## 1. Introduction

The restoration of endodontically treated teeth (ETT) is an important final step for successful root canal therapy. Excessive loss of tooth tissue, especially in the mesio-occluso-distal (MOD) cavity, and dentin dehydration may make the tooth prone fracture after the final restoration of ETT [1]. Therefore, it is important to strengthen teeth intracoronally to prevent fractures. The literature concerning how bonded restorations can fortify weakened teeth is inconsistent [2–5].

Many in vitro studies have shown that directly bonded restorations can fortify the tooth against fracture [2, 3]. Given the recent developments in composite materials, it is possible to create conservative and highly esthetic restorations. One such improvement is the bulk-fill composites. Their features include reduced volumetric shrinkage and increased cure depth, which allow for single incremental placement using layers up to 4 mm in thickness [6]. The potential advantage of these bulk-fill composites is that they can make clinical techniques simpler and faster, particularly in ETT with wide cavity restoration.

Fibers have further improved the properties of composite restorations. Ribbond (Ribbond, Seattle, WA, USA) is a polyethylene fiber which increases the flexural features of the composite restorations, and they allow efficient force transmission [7]. Previous studies showed that polyethylene fiber had a stress altering effect and inserting fiber under the composite restorations increased the fracture strength of endodontically treated teeth [8–10].

Teeth are continuously subjected to stress during mastication, swallowing, and parafunctional habits [11]. Little is known regarding the long-term clinical strength behavior of fiber insertion in bulk-fill composite restorations, especially in ETT. Clinical conditions are often simulated in vitro through thermal aging [12]. It is important to determine the effects of mechanical aging on fracture resistance of ETT with wide cavity restorations by simulating chewing.

The aim of this study is to determine the effects of different fiber insertion techniques and thermomechanical aging on the fracture resistance of endodontically treated mandibular premolar teeth restored with bulk-fill composite restorations.

The null hypotheses tested were as follows: (1) fiber insertion can increase the fracture resistance of endodontically treated mandibular premolar teeth restored with bulk-fill composite; and (2) thermomechanical aging can decrease the fracture resistance of endodontically treated mandibular premolar teeth restored with bulk-fill composite.

## 2. Material and Methods

In the present study, eighty human mandibular premolars of similar size (mesiodistal; 5.8 ± 0.3 mm, buccolingual; 7.4 ± 0.7 mm,) were used that had all been extracted for orthodontic reasons. As soon as they were extracted, the teeth were cleaned, the soft tissue remnants or other debris were removed, and the teeth were stored in distilled water. The teeth were then divided randomly into eight groups (n=10). Two control groups were planned. The first control group, Group IN (intact), and the second control group, Group IN-M (intact but subjected to thermomechanical aging), were not given any endodontic or cavity procedures. The teeth in the tested groups had endodontic treatment first. Diamond burs (G&Z Instrumente Gmbh, Lustenau Austria) at high speed with water cooling were used to prepare the access of cavities for the endodontic procedures. Then, after the cavity procedures, the pulp tissues were removed. The working length of each tooth was measured using a #15 K-file (Densply, Maillefer, Switzerland). An endodontic motor (X SMART, Densply, Maillefer, Switzerland) with TS1 and TS2 (One shape, Micro Mega, Besançon, France) was used to instrument the root canals. EDTA gel (Dia Prep Plus, Dia Dent, Chongju, Korea) was used to lubricate each instrument during the procedure, and 2 mL of 5.25% NaOCl was used to rinse out the root canals before and after instrumentation. Absorbent paper was used to dry out the canals, and then the canals were filled using gutta-percha (Micro Mega, United Kingdom) and root canal sealer (AH Plus Sealer, Dentsply De Trey, Konstanz, Germany) applied using cold lateral condensation. Preheated instruments were used to remove any excessive gutta-percha. Afterwards, all teeth were prepared with standardized mesio-occluso-distal (MOD) cavities. The thickness of the lingual and buccal walls of the standardized cavity was 2.5 ± 0.2 mm, while the distance from the base of the fissure to the gutta percha was standardized to 3 mm. Afterwards, 1 mm of gutta percha was removed from the top of the canal orifices and coated with a light-cured resin-modified glass-ionomer cement (Glass liner, Willmann & Pein, Barmsteadt, Germany). The gingival walls of the cavities were prepared to a distance 1.5 mm from the coronal aspect to the cementoenamel junction. Once MOD preparations were completed, the teeth were divided randomly into six different groups.

Table 1 shows tjat the materials that were used in the present study.

**Figure 1:**
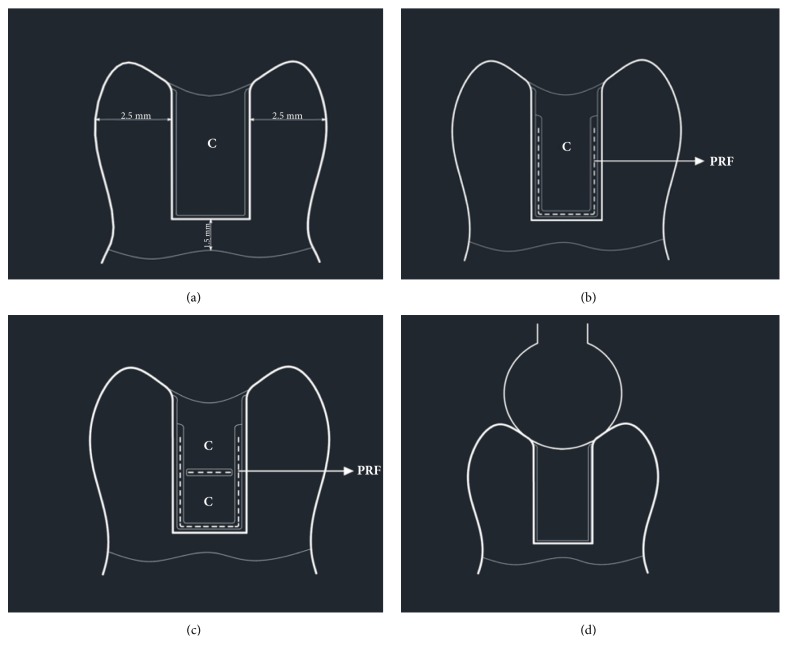
(a) Presentation of Group BF restored with a composite resin (C= Composite resin). The thickness of the lingual and buccal walls of the cavity was 2.5 mm; the gingival wall of the cavity was 1.5 mm from the coronal aspect to the cemento-enamel junction. (b) Presentation of Group PRF1 restored with a polyethylene reinforced fiber under composite resin (PRF=. polyethylene reinforced fiber). (c) Presentation of Group PRF2 restored with an additional polyethylene reinforced fiber. (d) A compressive force was applied with a stainless-steel ball parallel to the long axis of the teeth.

**Group Bulk-fill (BF):** Primer was applied to the cavities for 20 s (SE Primer; Kuraray, Tokyo, Japan), and then the cavities were dried carefully. Afterwards, the cavities were treated with SE Bond (Kuraray Medical Inc, Tokyo, Japan) and cured for 10 s with an LED light curing unit (LCU) (1000mW/cm^2^) (Valo, Ultradent Products Inc, South Jordan, UT). 3 mm bulk-fill resin composite (Estelite Bulk Fill Flow, Tokuyama, Japan) was used to restore the cavities and was polymerized for 20 s (Figure 1(a)).

**Group Polyethylene Ribbond Fiber 1** (**PRF1):** Primer and bonding procedures were applied the same as for Group BF. A layer of Estelite Bulk-Fill Flow was applied to the pulpal, lingual, and buccal cavity walls. Before curing, polyethylene fiber (width: 3 mm, length: 8 mm) (Ribbond; Ribbond Inc., Seattle, WA, USA) was cut and wetted with Clearfil SE Bond. The Ribbond was stored in a light-proof container prior to restoration. One piece of Ribbond was embedded into the flowable composite extended towards on the pulpal wall and 2/3 buccal and lingual walls and cured for 20 s. After curing, the cavities were restored with the same bulk-fill composite (Figure 1(b)).

**Group Polyethylene Ribbond Fiber 2** (**PRF2):** Clearfil SE Bond was used exactly as in the two previous groups. Again, the walls of the cavity were sealed with Estelite Bulk-Fill Flow. Then, Ribbond (width: 3 mm, length: 8 mm) was cut and wetted with Clearfil SE Bond. One piece of Ribbond was stored in a light-proof container prior to restoration. The Ribbond then was embedded into the flowable composite on the buccal, lingual, and pulpal walls and was cured for 20 s. Half of the cavities were filled with uncured bulk-fill composite; then, a second Ribbond piece (width: 3 mm, length: 3 mm) was embedded into the composite. Afterwards, all of the cavities were cured for 20 s. The rest of the cavities were restored with the same bulk-fill composite and cured again for 20 s (Figure 1(c)).

Thermomechanical aging was not applied to Groups BF, PRF1, and PRF2. Each tooth in these groups was placed into a self-curing polymethyl methacrylate resin (Imicryl SC, Imicryl Dental, Konya, Turkey) to a distance 1-1.5 mm below the cementoenamel junction (CEJ). The teeth were directly applied to a universal testing machine (AGS-X, Shimadzu, Tokyo, Japan) for fracture testing.

**Group Bulk-fill Thermomechanical Aging (BF-TMA):** The cavities were given the same restoration treatment as in Group BF. Then, the teeth were subjected to a thermocycling machine (SD Mechatronik Thermocycler, Feldkirchen-Westerham, Germany) (10,000 cycles, 5°C-55°C, 30 s of wait, and 10 s of transfer time). Following the thermocycling procedure, the teeth were placed in a self-curing polymethyl methacrylate resin to a distance 1-1.5 mm below the cementoenamel junction. During these procedures, it was ensured that the long axis of the tooth stayed parallel with the mold. Afterwards, the teeth were adapted to a chewing simulator (CS-4.2; SD Mechatronik, Feldkirchen-Westerham, Germany) and submitted to 50,000 load cycles and a frequency of 1.7 Hz to replicate an intermittent vertical load of 100 N on the restoration. During the test, the samples were submersed in distilled water.

**Group Polyethylene Ribbond Fiber 1 Thermomechanical Aging** (**PRF1-TMA):** The cavities were given the same restorative treatment as Group PRF1 and the same thermomechanical aging treatment as Group BF-TMA.

**Group Polyethylene Ribbond Fiber 2 Thermomechanical Aging** (**PRF2-TMA):** The cavities were given the same restorative treatment as Group PRF2 and the same thermomechanical aging as Group BF-TMA.

Once the restoration treatment was completed, all the samples were finished and polished using the diamond burs (FC Diamond, GZ Instrumente, Lustenau, Australia) and polishing discs (Soft-lex Dics, 3M ESPE, St. Paul, MN, USA). After these applications, the samples were fractured on the universal testing machine. The fracture test design was the form of a compressive force delivered parallel on buccal cusp tips to mimic the forces of centric occlusion, using a stainless-steel ball measuring 3 mm in diameter.

This ball was applied along the parallel axis of the teeth with the crosshead moving at a speed of 1 mm/min (Figure 1(d)). The data were recorded in Newtons (N).

Fracture surfaces were examined using a stereomicroscope at a magnification of 80X (SMZ 1000, Nikon, Japan) and were then categorized into two fracture modes:** (1) favorable,** meaning that the fracture was at the CEJ or above, or** (2) unfavorable,** meaning that the fracture was below the CEJ.

### 2.1. Statistical Analysis

The data were tested for normal distribution using the Skewness and Kurtosis Z-values and Shapiro–Wilk tests. Since the data (N) were normally distributed, two-way analysis of variance (ANOVA) and Tukey's honest significant difference (HSD) tests were used to compare the fracture resistance using a statistical software program, SPSS 15.0 (SPSS Inc., Chicago, IL, USA).

## 3. Results

The fracture strength values and statistical comparisons for each group are shown in Table 2. According to the results, the control groups (Group IN and Group IN-TMA) showed significantly higher fracture strengths than the tested groups (Groups BF, PRF1, PRF2, BF-TMA, PRF1-TMA, and PRF2-TMA) (*p*<0.05). No statistically significant difference was observed among the tested groups (*p*>0.05).

**Figure 2:**
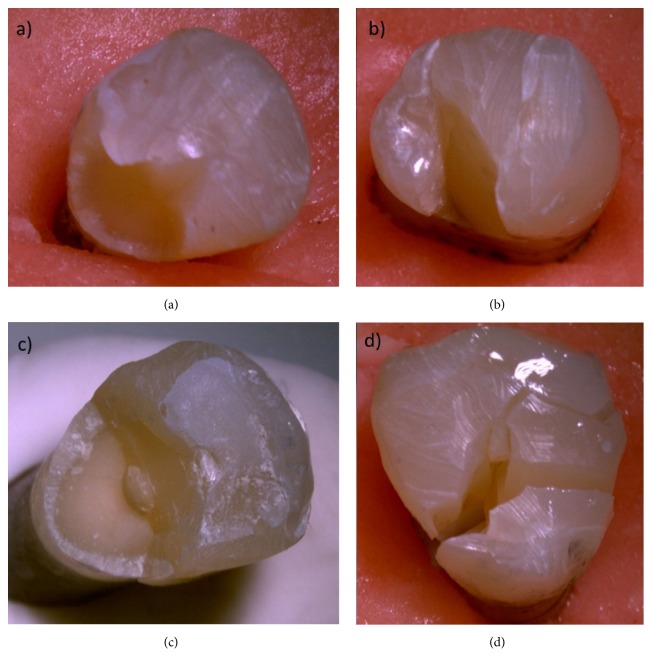
(a) Favorable fracture is seen in Group PRF1. (b) Favorable fracture is seen in Group PRF2. (c) Favorable fracture is seen in Group PRF2-TMA. The fracture line is above the cementoenamel junction. (d) Unfavorable fracture is seen in Group BF-TMA.

The failure modes for each group are displayed in Table 3. In terms of failure mode, the highest percentage of favorable fractures in the groups was observed in Group PRF1 (Figure 2(a)), followed by Group prf2 (Figure 2(b)) and Group PRF2-TMA (Figure 2(c)). Most of the unfavorable fractures were seen in Group BF-TMA (Figure 2(d)).

## 4. Discussion

In the present study, the effects of different fiber insertion techniques and thermomechanical aging on the fracture strength of ETT restored with bulk-fill composites were determined. The results showed that fiber insertion did not significantly increase the fracture strength of ETT restored with bulk-fill composite. In addition, thermomechanical aging did not decrease the fracture strength of the teeth used in the study. Therefore, both null hypotheses were rejected.

Inside the mouth, the posterior teeth are subjected to greater masticatory occlusal loads and are more prone to fracture than anterior teeth [13]. In the present study, root canal-treated mandibular premolar teeth with large MOD cavities were preferred to compare the strengthening and reinforcing properties of various restoration methods. In previous studies, universal testing machines have been used to produce a compressive load to the specimens by means of different metallic load (steel spheres and cylinders, wedge-shaped) devices [13–15]. It was determined that using a metal ball of a certain diameter is the best method to evaluate the resistance of premolars [16]. Therefore, in the present study, a vertical compressive loading (3-mm metal steel sphere) was applied to the teeth.

Various methods such as cyclic loading, water storage, or thermal cycling are commonly used to age the dental materials artificially [17, 18]. In this study, the teeth in Groups BF-TMA, PRF1-TMA, and PRF2-TMA were subjected to thermocycling and an artificial chewing simulator to simulate the oral environment and to better understand how these materials behaved under oral conditions. The International Organization for Standardization (ISO) recommends that thermal cycling between 5°C and 55°C is as an accelerated aging test [19]. This procedure imitates the range of temperatures created in the oral cavity by hot or cold drinks [20]. It is recommended that thermal cycling should range between 3,000 and 100,000 cycles, and it is suggested that 10,000 cycles represent one year of oral life [4, 21]. Therefore, to simulate one year of oral life, 10,000 thermocycles were applied in groups BF-TMA, PRF1-TMA, and PRF2-TMA in the present study.

Due to reduced elasticity, much less dentine and less water, it is believed that ETT treated teeth are more likely to fracture than vital teeth [1]. There is no consensus about the restoration types for ETT [4, 21]. Bulk-fill composite materials have started becoming popular for restoration ETT because these materials make it possible to build up cavities with a single increment placement to a depth of 4 mm. In addition, these materials are time-saving with less time chair-side and make clinical procedures easier [6]. Fiber technology has led to significant improvements in composite resins for dentistry in the areas of wider cavity preparations. Ribbond is a polyethylene fiber that is leno-woven and has an ultra-high-molecular-weight. Its high degree of elasticity dispersed the loads over a greater area resulting in a lower stress on the restoration and teeth [7].

In the present study, Ribbond did not increase the fracture resistance of the bulk-fill composite restorations. Atalay et al. [22] determined similar fracture strength results on endodontically treated maxillary premolar teeth restored with short fiber-reinforced composites (Ever X Posterior, GC, Tokyo, JAPAN) under nanohybrid composites, bulk-fill composites without fiber, and nanohybrid composites without fiber. They concluded that using short fiber-reinforced composites under a nanohybrid composite did not reduce the fracture strength. An earlier study by Belli et al. [10] reported the stress-relief effects of Ribbond (polyethylene fiber). They used a 3 mm wide, single piece of Ribbond under the hybrid resin composite with different insertion techniques. They found that fracture strength of endodontically treated mandibular molars with MOD cavities was significantly improved by the application of Ribbond. Hshad et al. [23] determined that inserting Ribbond under hybrid resin composites significantly improved the fracture resistance of endodontically treated mandibular premolar teeth. Different from our study, in all of these previous studies, conventional composites were used for restoring the cavities. The contradiction between these studies and our study could be the result of different types of composites and fibers. In the present study, Estelite Bulk-Fill Flow (low viscosity bulk-fill material) was used as a restorative material. The manufacturer claimed that Estelite Bulk-Fill Flow contains radical amplified photopolymerization (RAP) initiator coupled with camphorquinone (CQ), which increases polymerization rates and mechanical properties [24]. In addition, it contains a newly developed suprananospherical filler made of silica zirconia, which has excellent esthetic handling and mechanical properties [24]. These advanced structural properties of this material may have caused results different from the other studies.

To our best knowledge, thermomechanical aging was not used in the previous studies that were carried out to determine the effect of Ribbond on the fracture resistance of composites. In the present study, both thermal and mechanical aging were applied to the teeth in groups BF-TMA, PRF1-TMA and PRF2-TMA. When the fracture strength results were compared between the groups with or without thermomechanical aging, no significant difference was observed. In other words, thermomechanical aging did not affect the fracture strength results of the groups. However, in this study, a 100 N load was applied to the premolar teeth during the mechanical aging. However, in our opinion, higher mechanical loads could change these results.

When the fracture resistances of the tested groups were compared to those of the control groups, all of the tested groups demonstrated a reduced fracture resistance. Similar to our findings, Belli et al. [10], Khan et al. [25], and Sengun et al. [9] determined that there was a significant reduction in fracture resistance between the intact teeth and the teeth in the restorative groups.

In the present study, each group's fracture failure mode was also analyzed by stereomicroscopy. It was noted that unfavorable fractures were more common in teeth that had been restored with only bulk-fill composites (Groups BF and BF-TMA). Although Group PRF1 had favorable fractures, the number of favorable fractures was less than the number in Group PRF1-TMA, which had the thermomechanical aging procedure. In contrast, both the PRF2 and the PRF2-TMA Groups had favorable fractures. One possible explanation for this is the additional application of Ribbond between the composite increments. Additional application of Ribbond, together with the Ribbond applied to the cavity walls (buccal, lingual, and pulpal), may play a role as crack-stopping or crack-deflecting.

This study has some limitations. Even though fracture strength was studied, the biomechanical properties of the periodontium cannot be simulated as in the oral environment. The forces (100 N) in this study were applied in a continuous direction and speed, but the masticatory forces inside the mouth vary in force, speed, and direction. Further studies are necessary to determine how lateral and higher chewing forces affect the duration of restorations.

## 5. Conclusions

Despite the limitations of the study, the null hypotheses were rejected, and it is concluded that

- Both of the control groups demonstrated greater fracture strength than the tested groups.
- Fracture strength in endodontically treated mandibular premolar teeth with MOD preparations was not improved by the application of Ribbond beneath the bulk-fill composites.
- Fracture strength was not altered by thermomechanical aging in any of the groups.
- The fracture failure modes in Groups PRF1, PRF2, and PRF2-TMA mainly occurred in the enamel (favorable fracture), while in Group BF-TMA, they were mainly seen below the CEJ (unfavorable).
- Most of the fractures seen in Group PRF1 occurred in the enamel, but those in Group PRF1-TMA occurred mainly below the CEJ.
- While thermomechanical aging had a negative effect on the fracture mode results for Group PRF1-TMA, it did not change the favorable fracture results of Group PRF2-TMA.

## Figures and Tables

**Table 1:** Materials used in the present study and their compositions.

**Type of material**	**Lot. No**	**Manufacturer**	**Compositions**
Clearfil SE Bond	1D0056	Kuraray CO., LTD, Japan	Primer: MDP, HEMA, Hydrophilic dimethacrylate, *N,N*-diethanol-*p*-toluidine, water. Bond: MDP, Bis-GMA, HEMA, Hydrophobic dimethacrylate, CQ, *N,N*-diethanol-*p*- toluidine, silanized colloidal silica.

Estelite Flow Quick	235E07	Tokuyama Dental Corporation, Tokyo, Japan	Bis-MPEPP, TEGDMA, UDMA, Silica zirconia filler, silica titania filler, CQ, RAP initiator system

Estelite Bulk-Fill Flow	009E17	Tokuyama Dental Corporation, Tokyo, Japan	Bis-GMA, TEGDMA, Bis-MPEPP, Mequinol, Dibutyl hydroxyl toluene, UV absorber, spherical silica-zirconia filler, CQ, RAP initiator system

Ribbond	9512	Ribbond, Inc, Seattle, Washington, USA	Ultra–high molecular weight polyethylene, Homopolymer H-(CH_2_-CH_2_)n-H

**Table 2:** Mean fracture resistance of the groups and statistical differences among them.

**Groups**	**Thermo-mechanical Aging - (TMA)**	**Thermo-mechanical Aging + (TMA)**
	**Mean±SD** **(Newton)**	**Mean±SD** **(Newton**
**Group IN**	1351.4 (238.8) ^Aa^	1210.1 (318.5) ^Aa^

**Group BF**	736.8 (116.4) ^Ba^	788.7 (210.5) ^Ba^

**Group PRF1**	818.9 (166.1) ^Ba^	803.3 (78.1) ^Ba^

**Group PRF2**	821.9 (226.3) ^Ba^	832 (209.2) ^Ba^

Superscripts show the significant difference between the groups, while lower cases show the significant difference of columns **(*p*<0.005). SD=standard deviation.**

**Table 3:** The fracture modes and percentages of the groups.

	**Thermo-mechanical Aging (TMA) -**				**Thermo-mechanical Aging (TMA) +**			
**Groups**	**Favorable Fracture**		**Unfavorable Fracture**		**Favorable Fracture**		**Unfavorable Fracture**	
**Group IN**	6	60%	4	40%	5	50%	5	50%

**Group BF**	4	40%	6	60%	1	10%	9	90%

**Group PRF1**	8	80%	2	20%	2	20%	8	80%

**Group PRF2**	6	60%	4	40%	6	60%	4	40%

## Data Availability

The data used to support the findings of this study are available from the corresponding author upon request.
